# ThermoPCD: a database of molecular dynamics trajectories of antibody–antigen complexes at physiologic and fever-range temperatures

**DOI:** 10.1093/database/baae015

**Published:** 2024-03-19

**Authors:** Puneet K Singh, Razvan C Stan

**Affiliations:** Department of Basic Medical Science, Chonnam National University, Hwasun 58128, Republic of Korea; Department of Basic Medical Science, Chonnam National University, Hwasun 58128, Republic of Korea

## Abstract

Progression of various cancers and autoimmune diseases is associated with changes in systemic or local tissue temperatures, which may impact current therapies. The role of fever and acute inflammation-range temperatures on the stability and activity of antibodies relevant for cancers and autoimmunity is unknown. To produce molecular dynamics (MD) trajectories of immune complexes at relevant temperatures, we used the Research Collaboratory for Structural Bioinformatics (RCSB) database to identify 50 antibody:antigen complexes of interest, in addition to single antibodies and antigens, and deployed Groningen Machine for Chemical Simulations (GROMACS) to prepare and run the structures at different temperatures for 100–500 ns, in single or multiple random seeds. MD trajectories are freely available. Processed data include Protein Data Bank outputs for all files obtained every 50 ns, and free binding energy calculations for some of the immune complexes. Protocols for using the data are also available. Individual datasets contain unique DOIs. We created a web interface, ThermoPCD, as a platform to explore the data. The outputs of ThermoPCD allow the users to relate thermally-dependent changes in epitopes:paratopes interfaces to their free binding energies, or against own experimentally derived binding affinities. ThermoPCD is a free to use database of immune complexes’ trajectories at different temperatures that does not require registration and allows for all the data to be available for download.

**Database URL**: https://sites.google.com/view/thermopcd/home

## Introduction

Fever is an evolutionarily conserved innate response that contributes to survival during infections or other pathological conditions and that is generally restricted to a range of 38–40°C. After excluding the presence of infectious agents, fever of unknown origin, shown to be secondary to underlying cancers, is a clinically relevant concern (1). Different cancer models and associated treatments induce changes in the core body temperature of the patients (i.e. 37°C). The subsequent fever response stems from three causes. First, fever is a common side effect to immune checkpoint inhibitor (ICI) therapy (2). Second, neoplastic fever may be the culprit, as with most haematologic cancers (3). Last, neutropenic fever is a common reaction to chemotherapy (4). Notably, the tumour inflammatory milieu is itself warmer by up to 2°C than the surrounding healthy tissues. For instance, inner tumour temperatures are 1–2°C above the physiological core body temperature in lung, bladder, breast, skin and brain cancers (5).

Fever is thus an unavoidable aspect of cancer progression and of the immune responses to cancers, and its role on ICI therapy has yielded contrasting views. For instance, during tumour growth, signalling regulated by the PD-1/PD-L1 pathway is also associated with substantial inflammatory effects (6). As such, sustained fever associated with anti PD-1 mAb monotherapy appears to be a poor prognostic factor for patients (7). Further, inflammation can promote resistance to ICI in some cancer models (8). In turn, antipyretic medication has a marked adverse effect on ICI efficacy (5).

In addition, many types of autoimmune diseases include a component of the inflammatory response, with direct evidence indicating that the heat locally generated in inflammed tissue is up to 2°C higher than the average physiological temperature (9).

It is important therefore to address what the roles of inflammation and fever-range temperatures (38°C–40°C, 311 K–313 K) are on the structure and the activity of monoclonal antibodies relevant for the therapies of various cancers. A growing body of data indicates that the binding affinity of mature antibodies is enhanced at 40°C as opposed to the data obtained at 37°C *in vitro* (10), while *in silico* results suggest either positive (11) or negative effects (12) of higher temperatures on the activity of various antibodies binding to cancer or autoimmune targets.

Among the multitude of biophysical and biochemical techniques that are used for studying protein interactions, MD simulations provide the highest temporal and spatial resolution, with the additional advantage of accounting for context-dependency (e.g. temperature) of these processes. However, their key drawback is the computational cost, which generally prohibits large-scale and/or systematic analyses. It is important to note however, that there have already been a few initiatives to produce and analyse MD simulations of proteins in their native state, with an aim to describe their global flexibility that may be relevant for their function (13–17). While this area of research remains active, data obtained by independent groups can make comparative analyses difficult, due to different software packages and/or force fields used during the simulations. To the best of our knowledge, only three public databases of MD simulations provide general datasets for soluble proteins: MoDEL (13), Dynameomics (14) and ATLAS (16). Other relevant databases exist but focus on coarse-grained simulations of proteins, or on particular protein classes, such as those from SARS-CoV-2 (18). However, while ATLAS is available, MoDEL is no longer updated, and Dynameomics is currently inaccessible. Consequently, there is an unmet need to ensure that permanent access to each of such MD simulations is in place for users, in the form of unique digital object identifiers (DOIs).

Furthermore, despite the potential to use these data in, e.g. biotechnology or drug design, MD simulations of multiple proteins have been traditionally too expensive to run for long timescales (>100 ns), and prohibitively expensive when these proteins need to be run at multiple temperatures, for much longer simulation times and from different initial random seeds. Recently, two modalities have emerged that may be used to overcome the issue of cost: first, the advent of cloud-based supercomputing has greatly enhanced the resources and data sharing available to researchers (19) on one hand, while on the other hand, computational approaches relying on machine learning are moving forward the limitations of classical MD simulations into time scales that exceed the outputs obtained with coarse-grained dynamics (20, 21).

Here, we introduce the ThermoPCD database of MD trajectories for 50 protein complexes formed by therapeutic antibodies or autoantibodies against targets relevant for cancers and autoimmune diseases, respectively, at different pertinent temperatures. In addition, some MD trajectories describe only the antibodies or the antigens under same thermal conditions as the corresponding immune complexes. In ThermoPCD, each immune complex or antigen/antibody has been run, at a minimum, at three temperature points (310 K–312 K and at 313 K for most simulations) to 100 ns-long trajectories and up to 500 ns trajectories, in single to multiple independent runs. To each trajectory of a particular protein or protein complex at any particular temperature, we have assigned a unique and permanent DOI in the free-to-use Harvard Dataverse (https://dataverse.harvard.edu). ThermoPCD incorporates a tool for searches by antigen, antibody and/or PDB codes, in order to facilitate usability.

## Methods

### Database construction: structural data sources

Target antigens pertinent to cancers and autoimmune diseases were drawn in part from previous publications (22, 23). We have further filtered the results to focus on pathological conditions where fever was documented to have a clinical role, and where crystal structures were available with monoclonal antibodies (mAbs). Immune complexes were identified and extracted from the Research Collaboratory for Structural Bioinformatics (RCSB) Protein Data Bank (PDB) database, with the majority of the structures having a cutoff in the X-ray resolution of ≤3 Å. Most of the selected experimental protein structures are monomers, while few are functional as multimers. All antigens are human, unless otherwise specified.

An overview of the structures currently present in ThermoPCD is shown below in Table 1.

**Table 1.** PDB codes, descriptors, number of random seeds and lengths of simulations used in this study (as of March 2024)

PDB	No. of seeds	Description	Duration (ns)
6AL5^a^	1	CD19 (N138Q) in complex with Fab of mAb B43	100
3PP4	1	CD20 in complex with obinutuzumab	100
5VL3	1	CD22 in complex with Fab of Epratuzumab	100
5 TL5	1	CD27 in complex with M2177 mAb	100
1YJD^a^	1	CD28 in complex with 5.11A1 mAb	200
4CMH	1	CD38 in complex with SAR650984 mAb	100
1I9R	1	CD40L in complex with 5C8 mAb	100
7D4B	1	CD137 (4–1BB) in complex with llama single-domain antibody	100
4OD2	1	DR5 in complex with Fab of apomab	100
4ZQK	3	PD-1 in complex with ligand PD-L1	100, 200, 500
7E9B	1	PD-1 in complex with HLX10 mAb	100
6J14	1	PD-1 in complex with GY-14 mAb	100
7WSL	1	PD-1 in complex with dostarlimab	100
5GRJ	1	PD-L1 in complex with avelumab	100
5GGT	1	PD-L1 in complex with Fab of BMS-936 559 mAb	100
5X8L	1	PD-L1 in complex with atezolizumab	100
5XJ4	1	PD-L1 in complex with durvalumab-scFv	100
7DV4	1	CTLA-4 in complex with VH domain of 4003–1 mAb	100
5GGV	1	CTLA-4 in complex with tremelimumab Fab	500
6RP8	1	CTLA-4 in complex with ipilimumab Fab	500
1S78	1	ErbB2 (HER2) in complex with pertuzumab	100
1YY9	1	ErbB2 (HER2) in complex with Fab of cetuximab/Erbitux	100
5DK3 (mAb)	1	Pembrolizumab (Keytruda), IgG4 S228P anti-PD1 antibody	100
6YE3	1	IL-2 in complex with a Fab fragment of UFKA-20 mAb	100
4ZS7	1	IL-6 in complex with mAb 68F2	100
3HMX	1	IL-12 in complex with Fab of ustekinumab	100
5FB8^a^	5	IL-16 in complex with 14.1 mAb	100, 200
7WKX	1	IL-17A in complex with HB0017 mAb	100
2VXT	1	IL-18 in complex with 125–2 H mAb	100
3D85	1	IL-23 in complex with Fab of 7G10 mAb	100
3WD5	1	TNFα in complex with Fab of Adalimumab	100
4G3Y^a^	5	TNFα in complex with Fab of Infliximab	200
5VL3	1	CD22 d1-d3 in complex with Fab of Epratuzumab	100
1ADQ	1	Human IgM RH in complex with IgG autoantigen	100
2XWT^b^	5	TSH receptor in complex with autoantibody K1-70 mAb	200
3G04^a^,^b^	5	TSH receptor in complex with autoantibody M22 mAb	200
6BFQ	1	GM-CSF in complex with autoantibody 4D4	100
6BFS^b^	3	GM-CSF in complex with auto-antibody F1	100
6BFS (antigen)	2	GM-CSF	100
5Y9J	1	BAFF (CD257) in complex with belimumab	100
6FXN	1	BAFF (CD257, oligomerized) in complex with belimumab	100
3UX9	1	IFN alpha-1/13 in complex with α1bScFv01 mAb	100
2Y5T	1	Autoantibody CIIC1 in complex with triple-helical C1 peptide	100
5OCY	1	Autoantibody ACPA E4 in complex with CII-C-48-CIT peptide	100
5OCX^b^	3	Autoantibody ACPA E4 in complex with CII-C-13-CIT peptide	100
5OCX (antigen)	2	CII-C-13-CIT peptide	100
5MV4^b^	2	ACC1 Autoantibody in complex with citrullinated CII616-639	100
5MV4 (antigen)	2	Citrullinated CII616-639 peptide	100
6SF6^b^	3	COMP-epitope P6 in complex with 15A mAb	100
6SF6 (antigen)	2	COMP-epitope P6 peptide	100

a Data at 313 K are not available.

b Free binding energy calculations are available.

Most simulations not only comprise antibody:antigen complexes, but we also performed simulations on full-length antibodies alone (pembrolizumab, PDB: 5DK3) or on antigens with or without an antibody bound (PDB: 5MV4, 5OCX, 6BFS, 6SF6). Because of its clinical importance, we also produced structures of PD-1:PD-L1 complexes (PDB 4ZQK). Due to the relative ease of crystallization of antibody Fab fragments, and therefore their preponderance in the RCSB database, antigens in complexes with Fabs are predominant in our datasets. Importantly, to date, there are very few structures of intact, full-length IgG1 or IgG2 antibodies (24), but we made use of a full-length IgG4 structure (pembrolizumab) for comparisons with other immune complexes that contain PD-1 (PDB: 7E9B, 6J14, 7WSL, 5GRJ in our data).

ThermoPCD includes not only antigen targets, mainly cytokines and chemokines, but also PD-1, PD-L1 and CTLA-4 relevant for immunotherapy, in complexes with some of the most commonly used blockbuster ICI. Data also include free binding energy calculations for 6/45 existing entries.

### Preparation of protein structures

A detailed experimental protocol is present in the Supplementary Information 1. Briefly, unless otherwise noted, water and ligand molecules were removed from immune complexes to ensure protocol uniformity. When missing residues were found, MODELLER v10.1 was used to reconstruct the structures as loop regions (25). All-atom MD simulations were performed GROningen MAchine for Chemical Simulations (GROMACS2020) (http://www.gromacs.org/) using CHARMM27 (CHARM22 plus CMAP for proteins) force-field periodic boundary conditions. The structures were solvated in a truncated octahedron box of simple point charge water model. The solvated systems were neutralized with Na^+^ or Cl^−^ counter ions using the tleap program. Particle Mesh Ewald was employed to calculate the long-range electrostatic interactions. The cut-off distance for the long-range van der Waals energy term was 12.0 Å. The systems were then minimized at a maximum force of 1000.0 KJ/mol/nm by using 50 000 steps. The solvated and energy minimized systems were further equilibrated for 100 ps under NVT and NPT ensemble processes. In this work, we have deployed two thermostats: an initial Berendsen thermostat, due to its capability to equilibrate rapidly large protein complexes at the temperatures of interest, followed by the use of the V-rescale (Bussi–Donadio–Parrinello) thermostat for the MD production runs.

**Figure 1.**
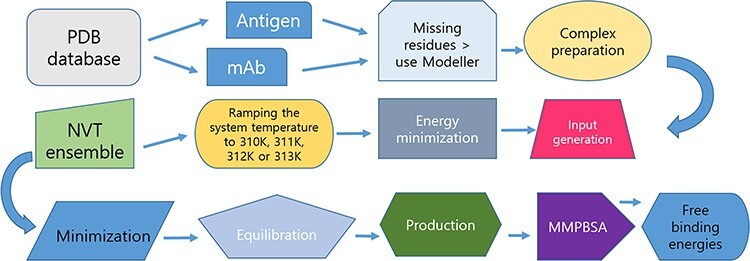
Protocol for data preparation and managing of the simulations.

A diagram of the workflow for producing the MD trajectories is presented in Figure 1.

## Results and discussion

### Database and web interface

All data are stored in the free and open Harvard Dataverse Repository (HDR, https://dataverse.harvard.edu) with full and free public access for downloading the datasets. Permanent DOIs are available for each individual file, ensuring citability and ease of use. All data are open and findable via search engines, and permanently stored, avoiding the issue of data unavailability as is the case with similar databases (26, 27). The experimental X-ray structure of each immune complex is taken as a reference and is also included on individual pages for all MD trajectories.

We have created the website ThermoPCD to HDR by using Google Sites. It provides a user-friendly interface where users can browse the whole database, avail of the hyperlinks to HDR data, and where an overview of all entries is managed. Information is organized and shown in different sections.

Updates and additions will be entered on both the ThermoPCD website and the corresponding HDR repository.

**Figure 2.**
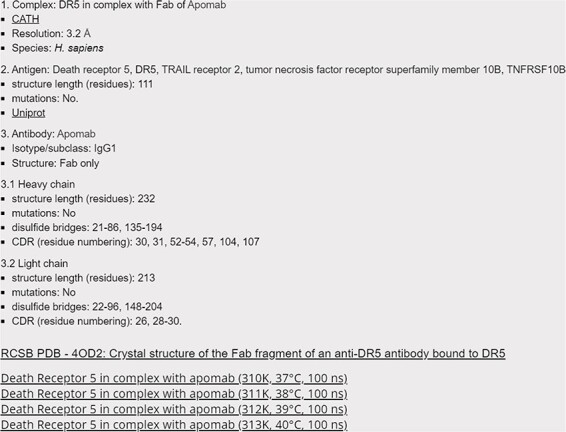
Screenshot of the 4OD2 entry (Death Receptor 5 in complex with apomab), with hyperlinks to CATH, Uniprot and RCSB databases, as well as to Harvard Dataverse for accessing the raw data.

All entries in ThermoPCD contain basic information on the immune complex (resolution, CATH entry, etc.) as well as on individual antibody and/or antigen (residues participating in CDR, location of disulphide bonds in antigens, etc.), as presented in Figure 2.

### Search for a protein

**Figure 3.**
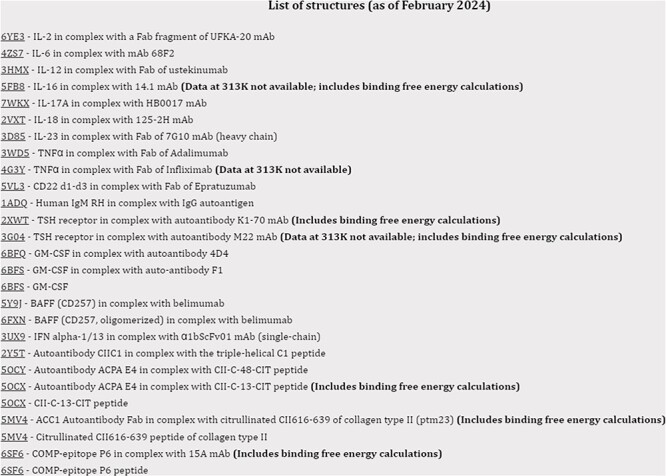
Screenshot of the index page with simulations for the structures relevant for autoimmune conditions.

Within each category, data can be called from the drop-down menu according to the name of the antigen. Users can further search for proteins by using their acronyms, part or entirety of their full names or the PDB codes in both ThermoPCD and the linked HDR repository using the search tool. The user can search for proteins with different combinations of filters. A list with all entries is present for each category, as shown in Figure 3.

### Protocols to enhance database use

To facilitate usability, we introduced protocols for interacting with data. For users interested in global parameters of interactions (e.g. buried surface areas between protein partners or changes in residues at the epitope:paratope interfaces) impacted by temperature increases, downloading the PDB files (a minimum of three structures for all data, starting with the initial structure(s) at 0 ns) allows for quick structural comparisons using e.g. PDBePISA (Proteins, Interfaces, Structures and Assemblies) server at https://www.ebi.ac.uk/pdbe/pisa. For users interested in determining other parameters of interactions between the immune partners, or of individual proteins, such as RMSD (root-mean-square deviation), RMSF (Root Mean Square Fluctuation), R*g* (radius of gyration), etc., we added a protocol with commands, on how to obtain this data (Supplementary Information 2).

Furthermore, we included a supporting file (Supplementary Information 3) with a complete protocol on how to determine binding free energy data from our files stored in Harvard Dataverse, based on an example from our database (RBD: 6RP8, CTLA-4 in complex with ipilimumab Fab). The user can follow the commands and use the scripts therein to explore how the binding free energies vary or not with changes in the simulation temperatures, and relate these data with changes in other parameters of interactions that may be affected (RMSF, etc.).

### Using data from ThermoPCD

#### Comparisons between structures

**Figure 4.**
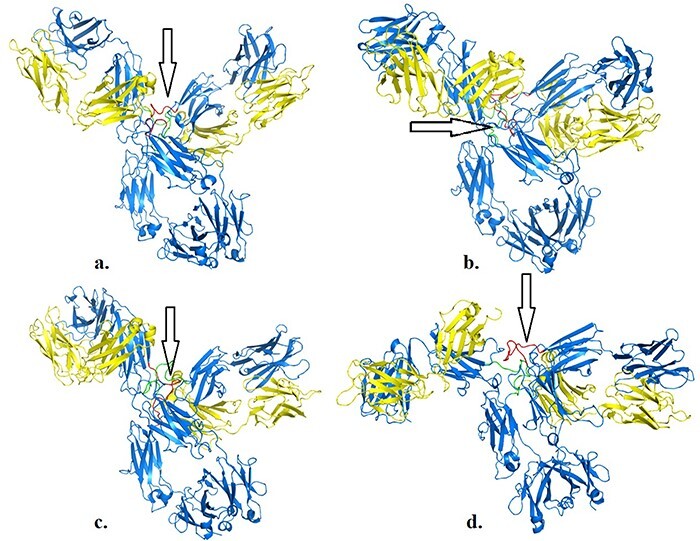
Structures of pembrolizumab (PDB 5DK3) after 100 ns at 310 K (a), 311 K (b), 312 K (c) and 313 K (d). Hinge regions are indicated by arrows.

We illustrate the workflow for assessing possible conformational changes at different temperatures in an antibody of clinical interest (PDB 5DK3, full-length pembrolizumab). This blockbuster mAb is important for immunotherapy in various cancer models, and therefore is important to determine whether physical factors such as temperature may impact its activity. Pembrolizumab is a very compact molecule due to the presence of a short hinge region between each Fab domain and Fc (24). As such, Fab domains are about 2 nm closer to each other and also closer to Fc, than corresponding IgG1 structures (Figure 1a and b in reference 24). We asked whether, from a structural viewpoint, temperatures attained during fever or acute inflammation may induce conformational changes in this molecule. Based on a first set of simulations (https://sites.google.com/view/thermopcd/cancers/pembrolizumab), we made use of the structures obtained after 100 ns, to determine the extent of movements of the Fab domains with temperature. Using PyMol (https://pymol.org/2), we prepared each pembrolizumab structure by highlighting the hinge regions (green and red, respectively, for each heavy chain). For comparison, we superimposed the structures obtained at 310 K and at 313 K with the original crystal structure, thus yielding RMSD of 0.36 nm and 1.52 nm, respectively. This increase in RMSD can be explained by visualizing Figure 4, whereby enhanced mobility of Fab domains is apparent from 310 K to 313 K. This behaviour at higher temperatures is similar to more independent domain motions and interactions that are known for IgG1 antibodies. Importantly, all the structures here used had the S228P mutation that is known to not only facilitate interchain disulphide-bond formation, but also to restrict Fab conformational changes (24), the latter of which appear to be relaxed on increasing temperature into a pathological range. Limiting or enabling these conformational changes in IgG4 antibodies may be important as these molecules have, on average, lower binding affinities, compared to IgG1 and IgG2 isoforms (24).

#### Assessing temperature-dependent changes in binding free energies in immune complexes

**Figure 5.**
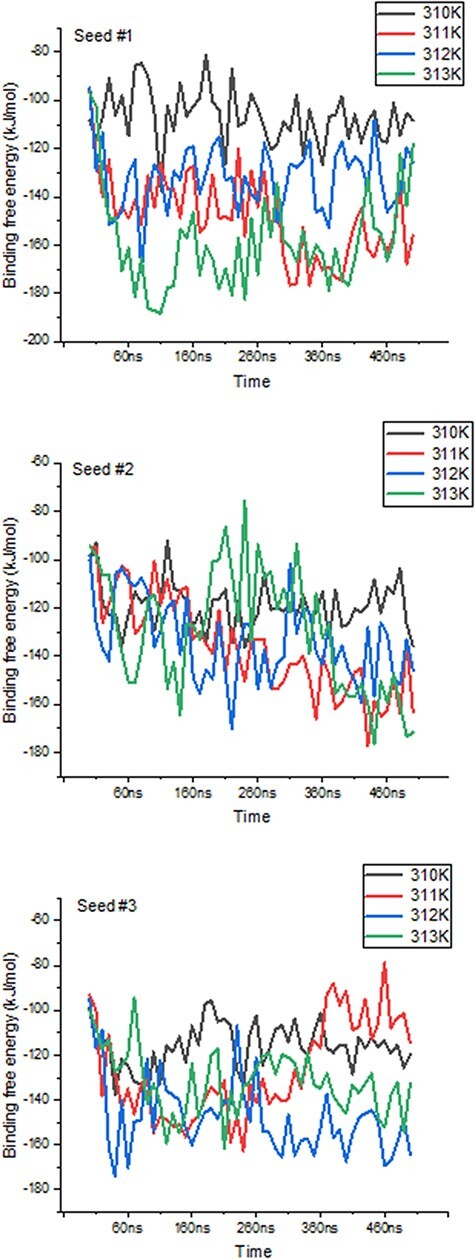
Variations in binding free energies between PD-1 and PD-L2 under different temperatures. Values obtained every 10 ns throughout the 500 ns-long simulations, for each seed and temperature point.

We further highlight ThermoPCD data as a means to estimate the binding free energies between partners of various immune complexes, under different thermal conditions. Using the example of human of PD-1/PD-L2 complex (PDB 6UMT), it is possible to monitor the changes in binding free energies from three random seeds, for a duration of 500 ns each, as indicated in Figure 5. The averages (in kJ/mol) of these binding free energies with temperatures were −114.2 ± 6.4, −132 ± 2.9, −148 ± 9.2 and −126.3 ± 8.1, from 310 K to 313 K, respectively. The increase in the binding free energy from 37°C to 39°C, by about 30%, between these important inhibitors of the immune response during cancer progression may, in part, shed light on the positive role of inflammation-range temperatures for tumour cells, and not only for the activity of T-cells, as previously shown (28). Further, it is possible that at the highest temperatures (i.e. 40°C), the structures of PD-1 and PD-L2 may be affected, to the extent that the binding interface is reduced, leading to a loss in favourable binding free energy.

#### Code availability

No custom code was used for the creation of this database and of the interface website. All software deployed is open source, including Modeller (25), Gromacs (29), AmberTools23 (30), PyMol (31) and ChimeraX (32).

#### Importance

Besides an interest in the role of febrile and inflammation temperatures on the formation of antibody:antigen complexes, our data may be used for other purposes. For instance, ensembles of the structures generated may be deployed to enhance docking performance of small ligands, as previously shown (33), to identify new druggable pockets in proteins or to detect patterns of occurrence of the amino acids at these protein interfaces, as noted before (34). Another interesting avenue for research may be in determining the role of fever in modulating the affinity of autoantibodies in autoimmune disorders, of which we produced structures for eight relevant immune complexes. Similarly, whether high temperatures have a role in exposing new epitopes from self-antigens through molecular mimicry, possibly following high-fever episode(s) caused by infections with, e.g. *Streptococcus pyogenes* (35), remains undetermined, and we have added simulations for four such immune complexes to test this hypothesis. Further, understanding the conformational flexibility of unbound antibodies that may be caused by physiological or pathological temperatures may be important in delineating new determinants of their binding affinity.

### Current and future development

We are planning to include additional random seeds in ThermoPCD database for existing entries, particularly of immune complexes used in immunotherapy. We are currently performing similar MD simulations on the antibodies from our datasets, at similar temperatures to those employed for respective immune complexes, and on different isolated antibody isotypes (IgG1: PDB 1IGY, 1HZH; IgG2: PDB 4L4J; IgG3: PDB 4HDI; IgG4: PDB 6GFE).

Future work will add MD trajectories of immune complexes, including of separated antigens and antibodies, that are relevant for fever-causing viral, protozoal and bacterial pathogens. In so doing, we seek to provide a platform that will aid in establishing the roles of physiological and pathological temperatures on the structural stability and activity of antibody:antigen complexes.

## Conclusions

Chronic inflammation may precede or accompany tumour progression and resistance to treatments (36), whereas acute inflammation, also called ‘therapeutic inflammation’ may assist in the maturation of dendritic cells and in antigen presentation, leading to more vigorous anti-tumour immune responses (37). Understanding the role of fever and inflammation-range temperatures is important for delineating the efficacy, or lack thereof, of, e.g. therapeutic antibodies. ThermoPCD contains a unique, well-curated and thoroughly linked database of protein conformations relevant for these conditions, at pertinent temperatures. Importantly, ThermoPCD database relies on experimentally determined structures as starting points for the MD simulations. This work does not address possible conformational changes at standard room temperature (22°C/295 K) or the common assay temperature for antibody:antigen interactions (25°C/298 K). The relation between temperature-dependent conformational motions in immune complexes will allow the probing of protein dynamics under clinically relevant thermal conditions for some of the most common cancers and autoimmunity targets.

## Supplementary Material

baae015_Supp
