# Supplementary material for: ThermoPCD: a database of molecular dynamics trajectories of antibody–antigen complexes at physiologic and fever-range temperatures
Source: Database (Oxford). 2024 Mar 19;2024:baae015. doi: 10.1093/database/baae015 (PMC10950042; doi:10.1093/database/baae015)
Supplement: baae015_Supp [file baae015_supp.zip › suppl_data/Supporting file 1. Computational methods.docx]

MD Simulation Process

Simulations were conducted with Gromacs2020 (http://www.gromacs.org/) using CHARMM27 (CHARM22 plus CMAP for proteins) force field periodic boundary conditions. The structures were solvated in a truncated octahedron box of Simple point charge water model. The solvated system was neutralized with Na+ or Cl− counter ions using the tleap program. Particle Mesh Ewald was employed to calculate the long-range electrostatic interactions. The cut-off distance for the long-range van der Waals energy term was 12.0 Å. The system was then minimized at a maximum force of 1000.0 KJ/mol/nm by using 50,000 steps.

The solvated and energy minimized systems were further equilibrated for 100 ps under NVT and NPT ensemble processes.


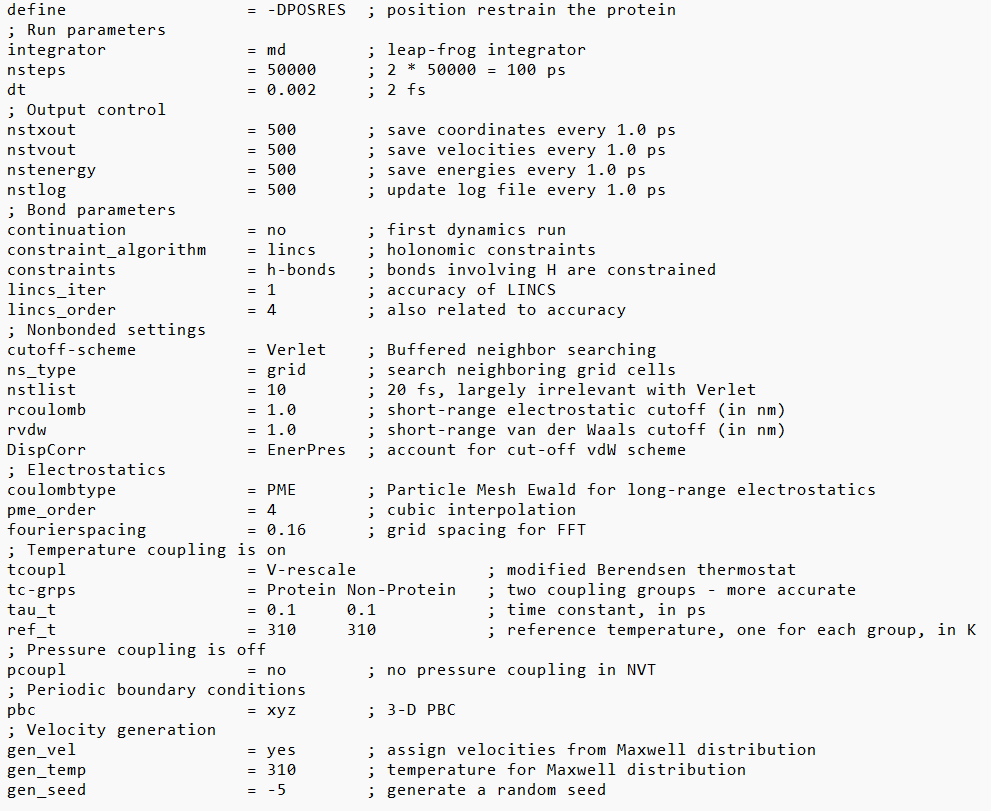
Find the mdp file for NVT (Constant temperature, constant volume) equilibration in the code box below. This mdp file is used for an equilibration in the NVT ensemble. This is usually required to bring the system to the desired temperature.

In the first line, we apply position restraints to the protein via the define=-DPOSRES keyword. Position restraints are contained in the posre.itp file which is created via the gmx pdb2gmx module, and it allows us to equilibrate the solvent around the protein without causing significant structural changes in the protein.

Then we have a section dedicated to the Run parameters


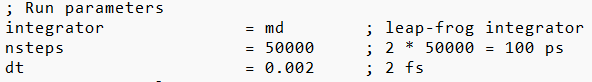


Here we specified:

1. The type of algorithm we want to use to solve the equations of motion. The leap-frog integrator was selected in our case (integrator=md)

2. The duration of the simulation is given by the time step we select (dt) as well as the number of steps to perform (nsteps). In this specific case, we selected a time step of 2 fs (dt=0.002) and we decided to perform 50000 steps (nsteps=50000). The overall simulation time is given by the time step multiplied by the number of steps.

The following paragraph is about how frequently to save information to the output files.


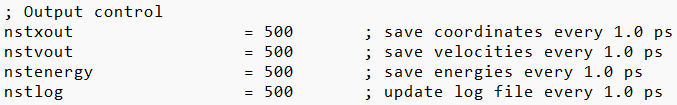


The nstxout=500 and nstvout=500 parameters represent the writing of the coordinates and the velocities to the trajectory file in the trr format every 500 time steps.

The nstenergy=500 parameter is needed for how often we want to save the energies in the edr output file. The nstlog=500 specifies how frequently to save information to the log file, which provides a summary of the run.

The next section:


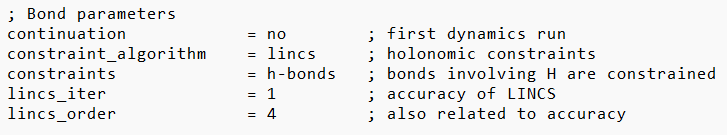


The purpose of this box is to mainly determine two things:

1. We are not continuing this simulation starting from another run (continuation=no). This is often the case for an NVT equilibration.
2. We want to constraint the bonds of the protein using the LINCS algorithm.


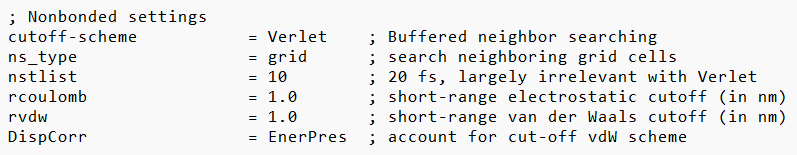
The next section

This paragraph contains the parameter related to the Electrostatic and Van der Waals (VDW) interactions. For this part, we have used a cut-off value in the range of 1 nm for both Electrostatic (rcoulomb = 1.0) and VDW (rvdw=1.0) interactions.


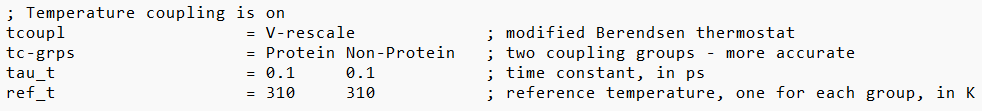
To bring the system to the desired temperature we employed V-rescale thermostat which is appropriate for a general equilibration [1].

We created separate groups consisting of the protein (Protein) and the rest of the system (non-protein) for the thermostat with the tc-groups parameter.

Finally, we choose the desired temperature (310K, 311K, 312K and 313K) for both groups with the ref_t parameter and the coupling constant with tau_t.

The next section

In an NVT simulation, the pressure of the system is not adjusted so the box of the system stays fixed (pcoupl=no)


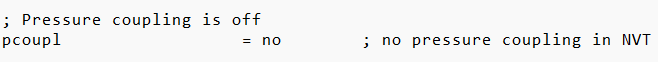


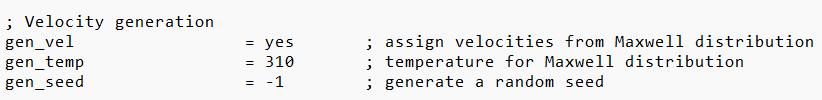
The last section of the NVT we assigned the initial velocities to our system.

The velocities is selected from a Maxwell-Boltzmann distribution at the desired temperature (310K, 311K, 312K and 313K) that we choose for the gen_temp parameter.

This is the point where we have the option to generate random seeds. We changed the gen_seed value to -1, -3, -5 and so on.

NPT (Constant temperature, constant pressure)


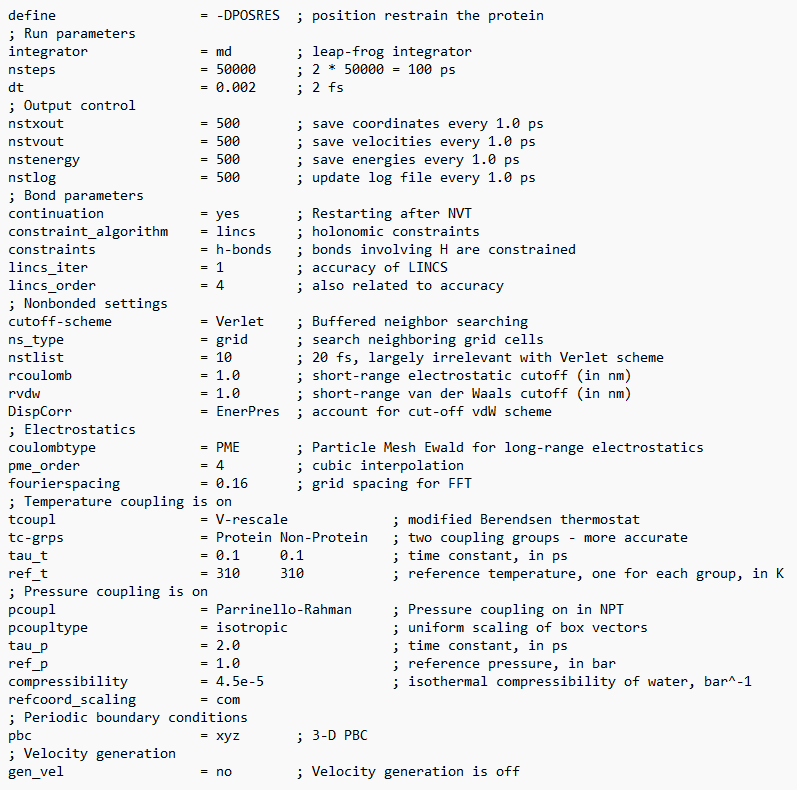
The NPT equilibration is used to bring the system to the desired pressure.

Most of the parameters in this file were already discussed in the NVT section. The main difference with respect to the previous file for the NVT equilibration are described below.


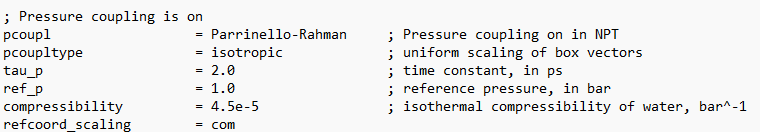
In this section the pressure coupling is on, and we adjusted the size of the box to reach the desired pressure value. We have used the pcoupl parameter as the Parrinello-Rahman.

We selected the ideal pressure value in bars with ref_p=1.0, and the time constant with tau_p=2.0. The size of the box is scaled uniformly in all three dimensions with pcoupltype=isotropic.

Production MD

This is generally a simulation within the NPT ensemble with no restraints on the protein.

Most of the parameters in this file were already discussed in the NVT and NPT section
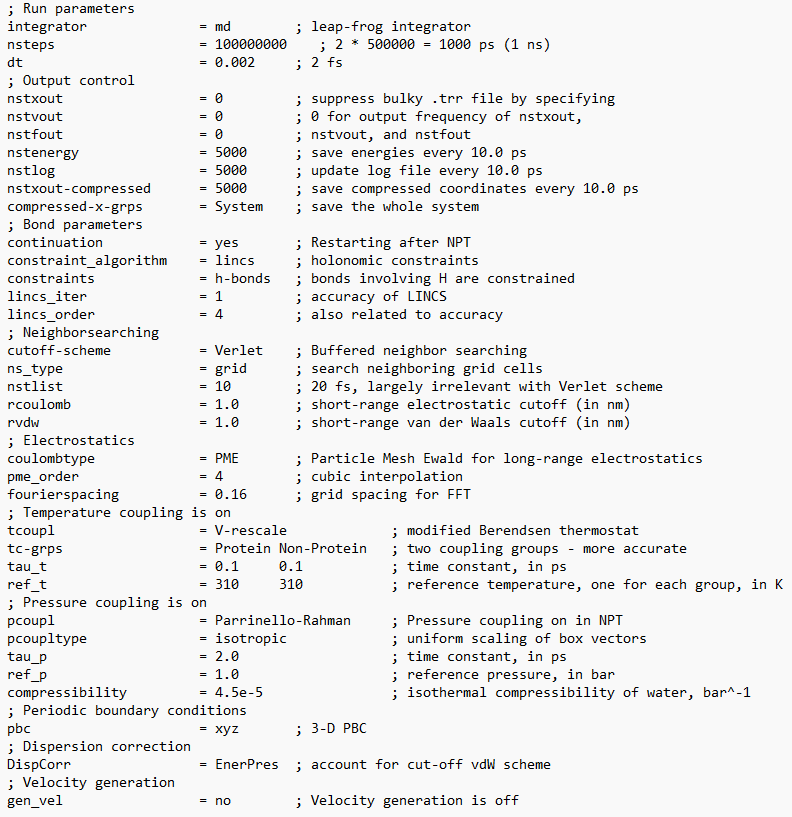
.

In the section run parameters we can enter the desired time for the run of simulation in nsteps. Here we have used 100000000 steps which equates to 100ns. We can increase or decrease the steps to get the desired length of simulation.

The output section differs from the NVT and NPT and is described below.


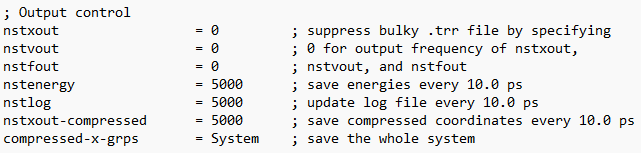


The output parameters that we previously saw (nstxout, nstvout) are all set to zero.

We have written the coordinated in the xtc file every 5000 steps (nstxout-compressed=5000). We have also specified saving the whole system by passing the compressed-x-grps=System parameter.

Supplementary references:

S1. Bussi G, Donadio D, Parrinello M. Canonical sampling through velocity rescaling. J Chem Phys. 2007 Jan 7;126(1):014101. doi: 10.1063/1.2408420.
