# Supplementary material for: ThermoPCD: a database of molecular dynamics trajectories of antibody–antigen complexes at physiologic and fever-range temperatures
Source: Database (Oxford). 2024 Mar 19;2024:baae015. doi: 10.1093/database/baae015 (PMC10950042; doi:10.1093/database/baae015)
Supplement: baae015_Supp [file baae015_supp.zip › suppl_data/Supporting file 2. Protocol to obtain parameters of interactions.pptx]

## Slide 1
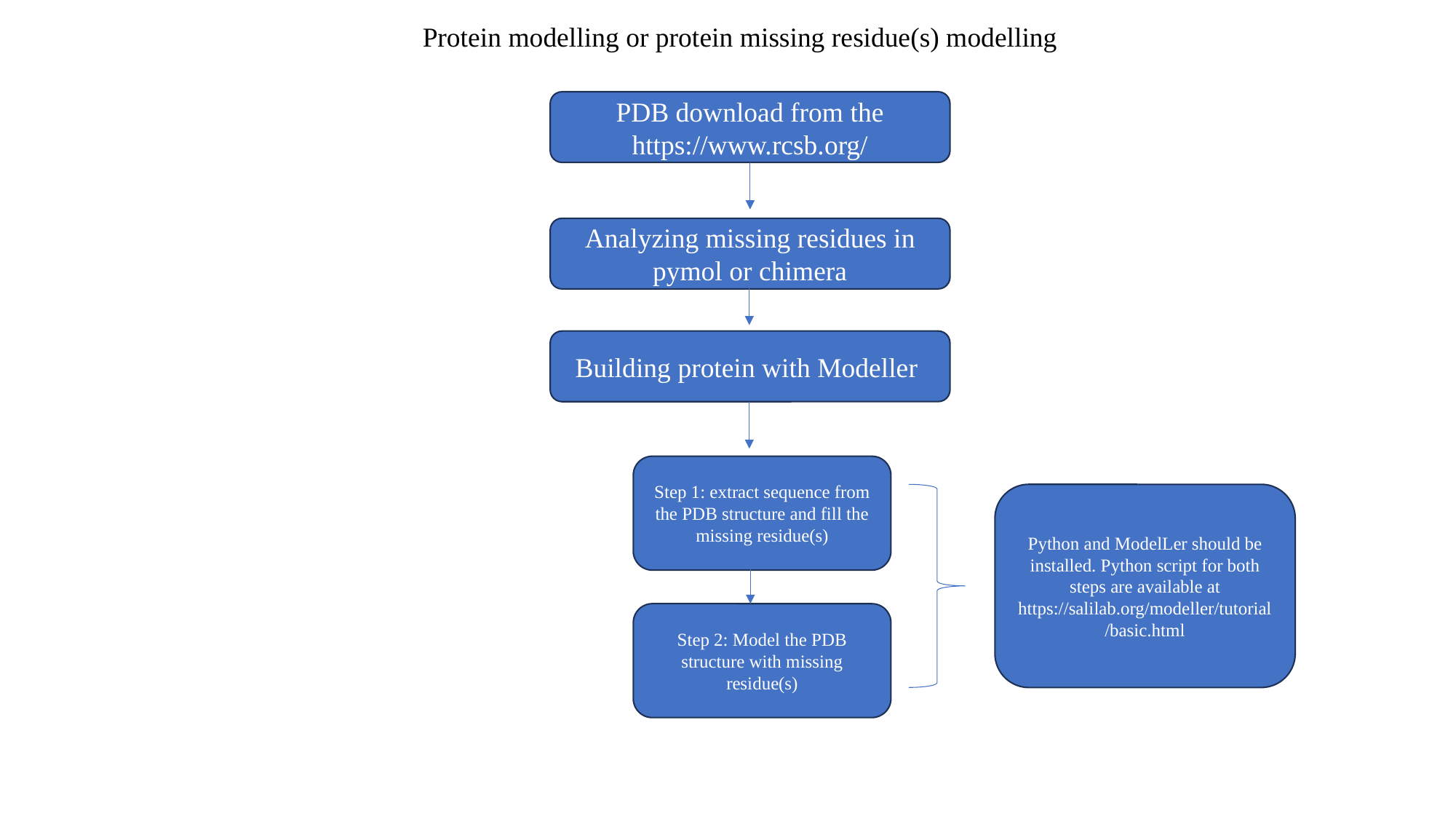

Protein modelling or protein missing residue(s) modelling
PDB download from the https://www.rcsb.org/
Analyzing missing residues in pymol or chimera
Building protein with Modeller
Step 1: extract sequence from the PDB structure and fill the missing residue(s)
Python and ModelLer should be installed. Python script for both steps are available at https://salilab.org/modeller/tutorial/basic.html
Step 2: Model the PDB structure with missing residue(s)

## Slide 2
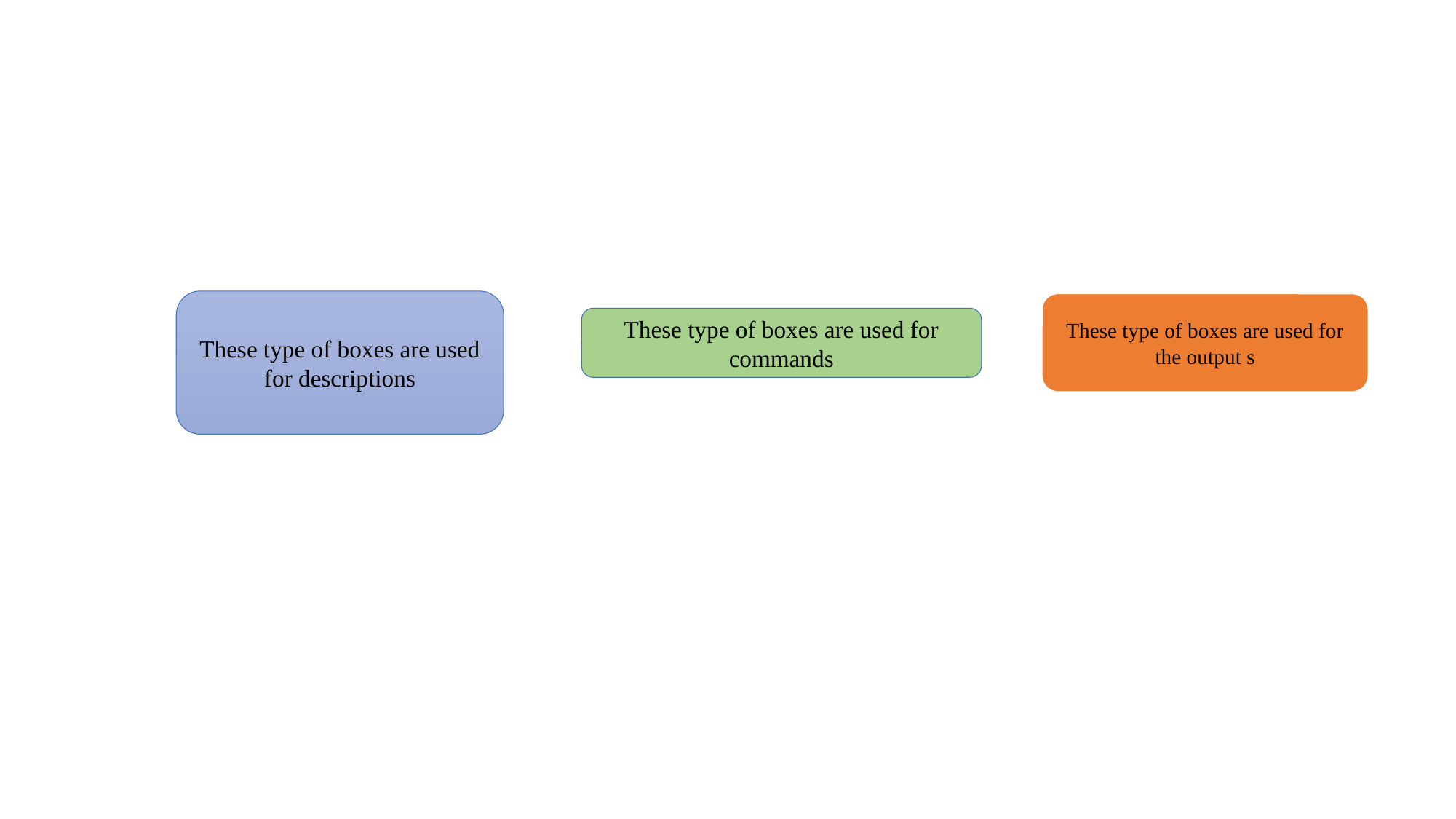

These type of boxes are used for descriptions
These type of boxes are used for the output s
These type of boxes are used for commands

## Slide 3
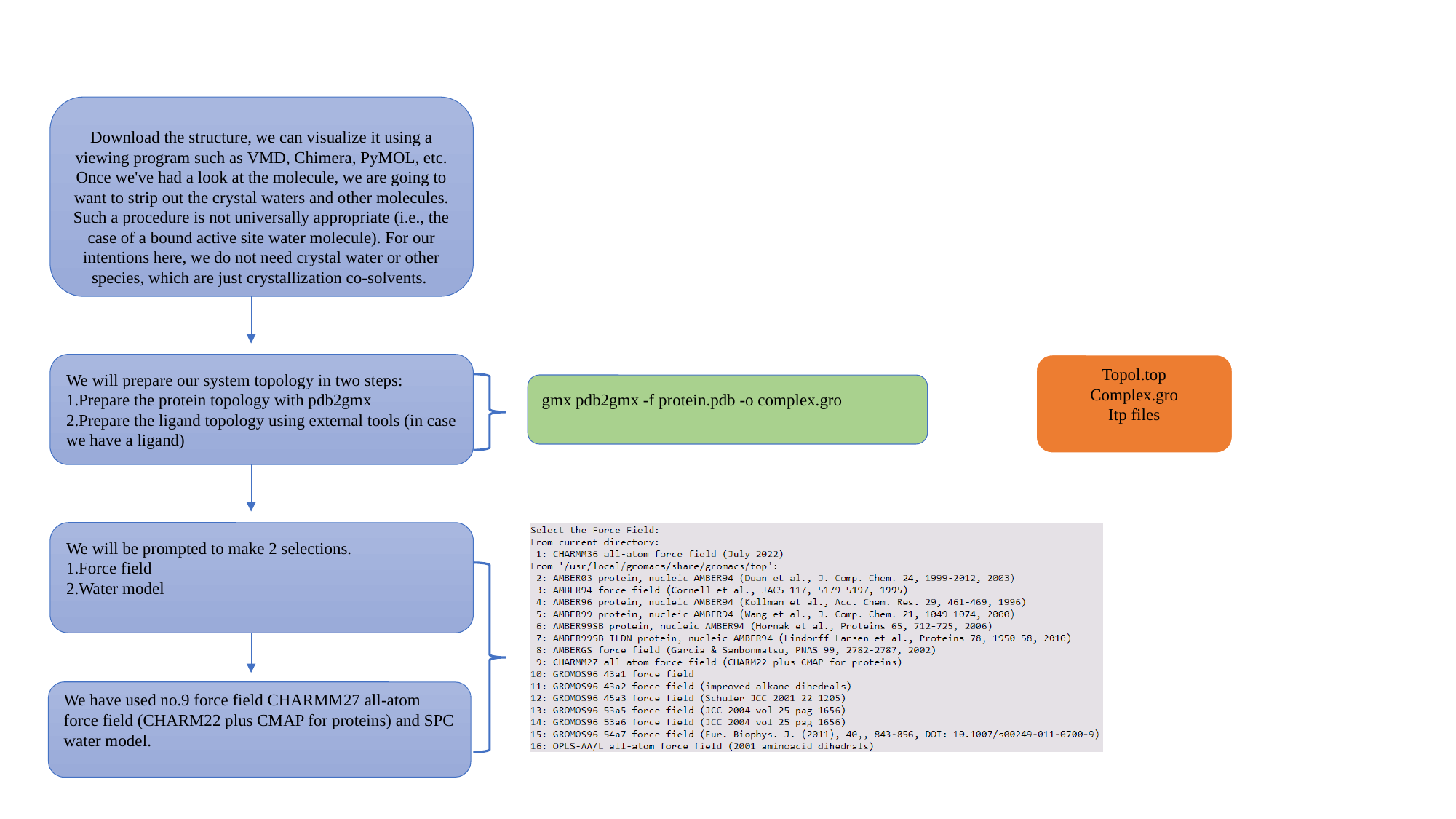

Download the structure, we can visualize it using a viewing program such as VMD, Chimera, PyMOL, etc. Once we've had a look at the molecule, we are going to want to strip out the crystal waters and other molecules. Such a procedure is not universally appropriate (i.e., the case of a bound active site water molecule). For our intentions here, we do not need crystal water or other species, which are just crystallization co-solvents.
We will prepare our system topology in two steps:
Prepare the protein topology with pdb2gmx
Prepare the ligand topology using external tools (in case we have a ligand)
Topol.top
Complex.gro
Itp files
gmx pdb2gmx -f protein.pdb -o complex.gro
We will be prompted to make 2 selections.
Force field
Water model
We have used no.9 force field CHARMM27 all-atom force field (CHARM22 plus CMAP for proteins) and SPC water model.

## Slide 4
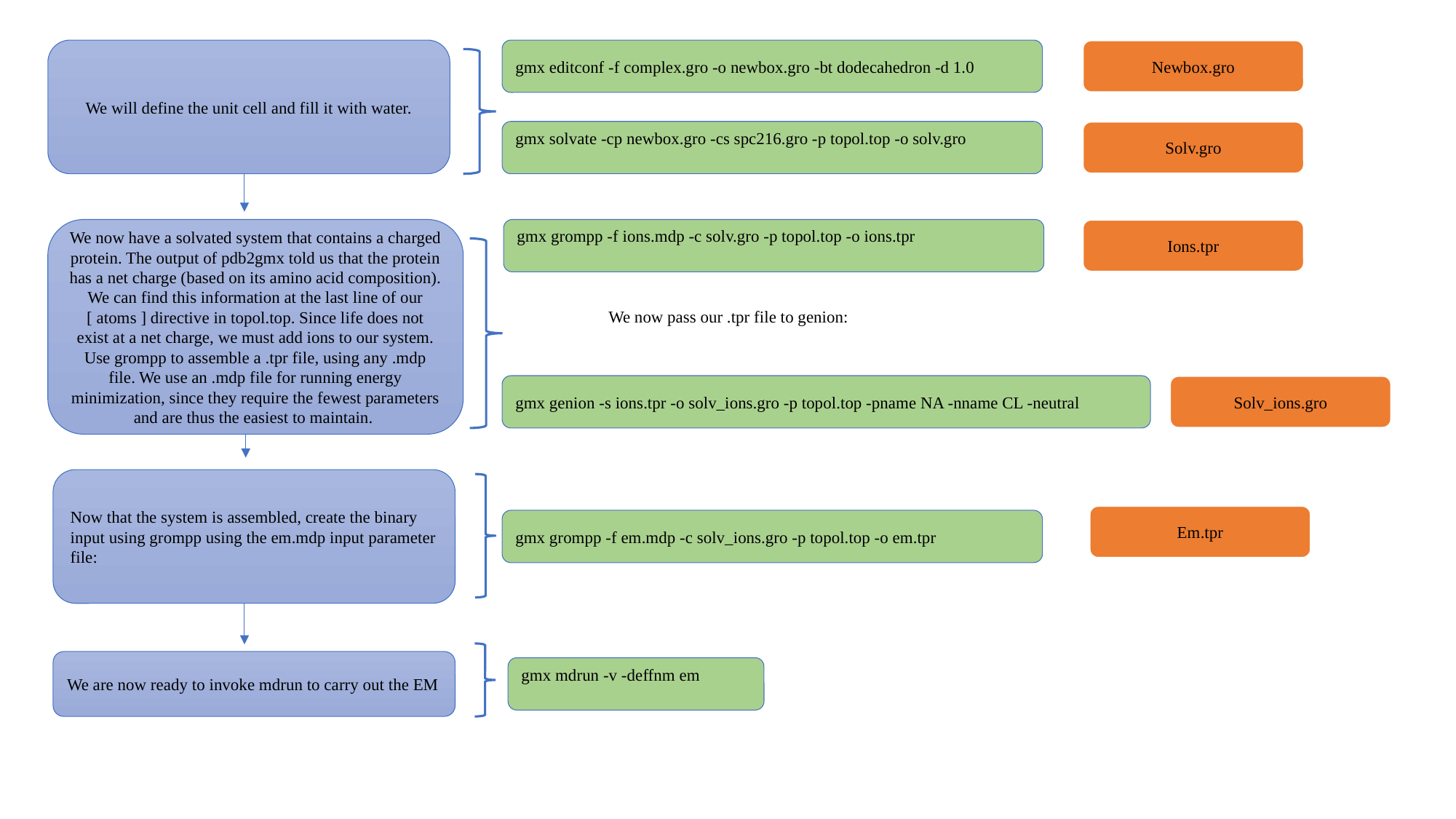

We will define the unit cell and fill it with water.
gmx editconf -f complex.gro -o newbox.gro -bt dodecahedron -d 1.0
Newbox.gro
gmx solvate -cp newbox.gro -cs spc216.gro -p topol.top -o solv.gro
Solv.gro
We now have a solvated system that contains a charged protein. The output of pdb2gmx told us that the protein has a net charge (based on its amino acid composition). We can find this information at the last line of our [ atoms ] directive in topol.top. Since life does not exist at a net charge, we must add ions to our system.
Use grompp to assemble a .tpr file, using any .mdp file. We use an .mdp file for running energy minimization, since they require the fewest parameters and are thus the easiest to maintain.
gmx grompp -f ions.mdp -c solv.gro -p topol.top -o ions.tpr
Ions.tpr
We now pass our .tpr file to genion:
gmx genion -s ions.tpr -o solv_ions.gro -p topol.top -pname NA -nname CL -neutral
Solv_ions.gro
Now that the system is assembled, create the binary input using grompp using the em.mdp input parameter file:
Em.tpr
gmx grompp -f em.mdp -c solv_ions.gro -p topol.top -o em.tpr
We are now ready to invoke mdrun to carry out the EM
gmx mdrun -v -deffnm em

## Slide 5
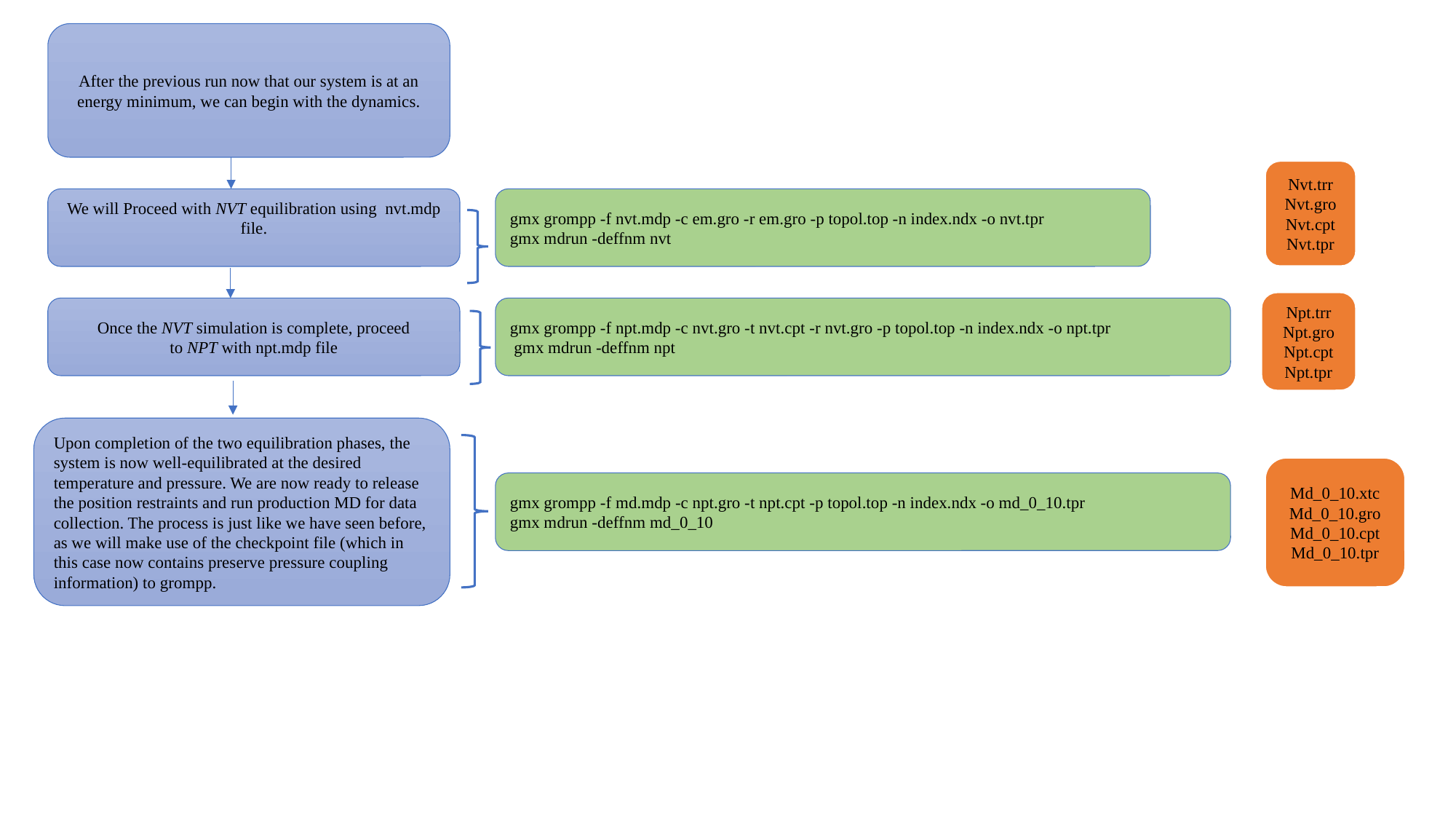

After the previous run now that our system is at an energy minimum, we can begin with the dynamics.
Nvt.trr
Nvt.gro
Nvt.cpt
Nvt.tpr
gmx grompp -f nvt.mdp -c em.gro -r em.gro -p topol.top -n index.ndx -o nvt.tpr
gmx mdrun -deffnm nvt
We will Proceed with NVT equilibration using  nvt.mdp file.
Npt.trr
Npt.gro
Npt.cpt
Npt.tpr
gmx grompp -f npt.mdp -c nvt.gro -t nvt.cpt -r nvt.gro -p topol.top -n index.ndx -o npt.tpr
 gmx mdrun -deffnm npt
Once the NVT simulation is complete, proceed to NPT with npt.mdp file
Upon completion of the two equilibration phases, the system is now well-equilibrated at the desired temperature and pressure. We are now ready to release the position restraints and run production MD for data collection. The process is just like we have seen before, as we will make use of the checkpoint file (which in this case now contains preserve pressure coupling information) to grompp.
Md_0_10.xtc
Md_0_10.gro
Md_0_10.cpt
Md_0_10.tpr
gmx grompp -f md.mdp -c npt.gro -t npt.cpt -p topol.top -n index.ndx -o md_0_10.tpr
gmx mdrun -deffnm md_0_10

## Slide 6
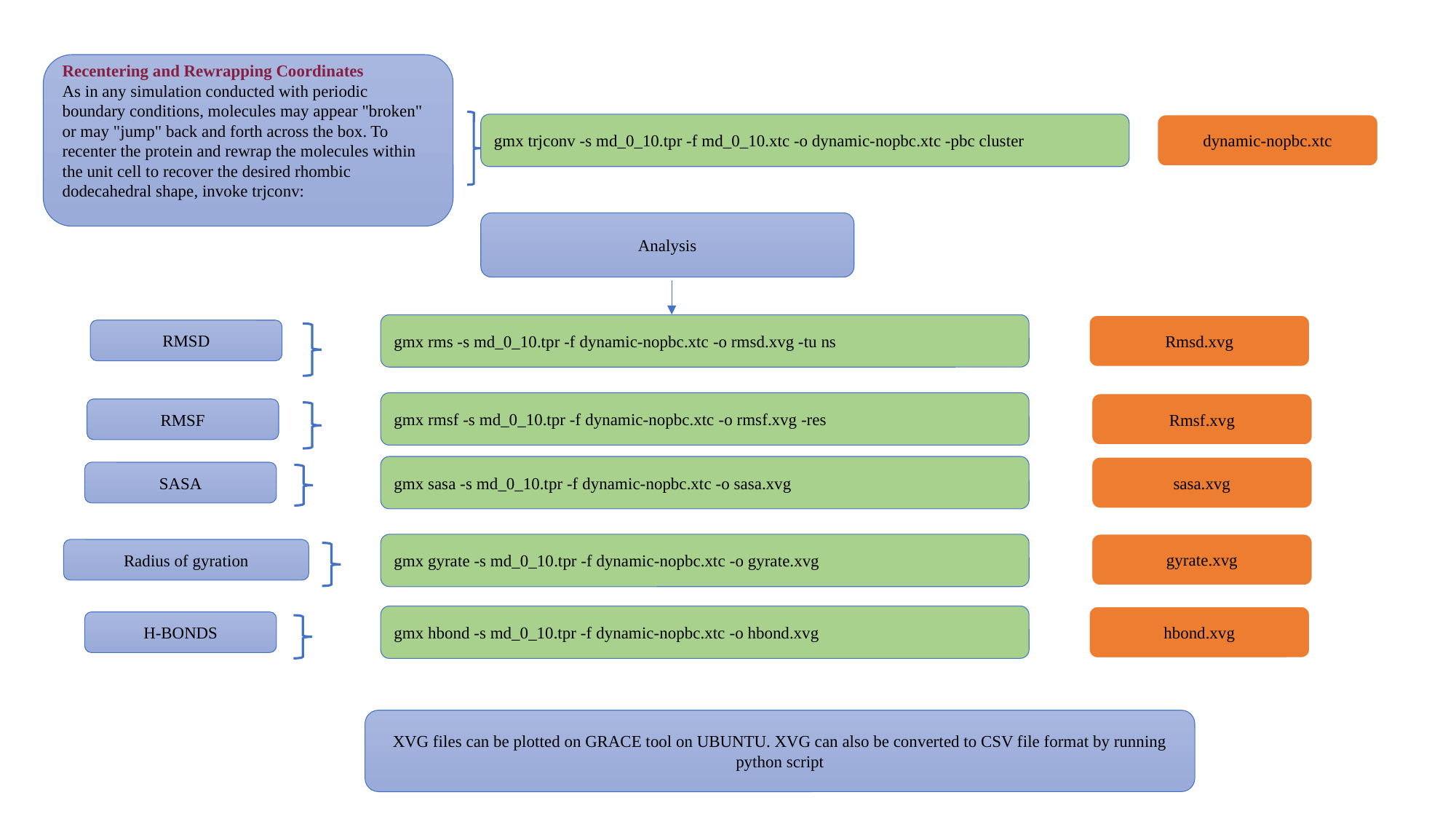

Recentering and Rewrapping Coordinates
As in any simulation conducted with periodic boundary conditions, molecules may appear "broken" or may "jump" back and forth across the box. To recenter the protein and rewrap the molecules within the unit cell to recover the desired rhombic dodecahedral shape, invoke trjconv:
gmx trjconv -s md_0_10.tpr -f md_0_10.xtc -o dynamic-nopbc.xtc -pbc cluster
dynamic-nopbc.xtc
Analysis
gmx rms -s md_0_10.tpr -f dynamic-nopbc.xtc -o rmsd.xvg -tu ns
Rmsd.xvg
RMSD
gmx rmsf -s md_0_10.tpr -f dynamic-nopbc.xtc -o rmsf.xvg -res
Rmsf.xvg
RMSF
sasa.xvg
gmx sasa -s md_0_10.tpr -f dynamic-nopbc.xtc -o sasa.xvg
SASA
gyrate.xvg
gmx gyrate -s md_0_10.tpr -f dynamic-nopbc.xtc -o gyrate.xvg
Radius of gyration
hbond.xvg
gmx hbond -s md_0_10.tpr -f dynamic-nopbc.xtc -o hbond.xvg
H-BONDS
XVG files can be plotted on GRACE tool on UBUNTU. XVG can also be converted to CSV file format by running python script
