# Supplementary material for: ThermoPCD: a database of molecular dynamics trajectories of antibody–antigen complexes at physiologic and fever-range temperatures
Source: Database (Oxford). 2024 Mar 19;2024:baae015. doi: 10.1093/database/baae015 (PMC10950042; doi:10.1093/database/baae015)
Supplement: baae015_Supp [file baae015_supp.zip › suppl_data/Supporting_file_3._Protocol_to_obtain_binding_free_energy_data...docx]

We will use [PDB **6RP8**](mailto:https://www.rcsb.org/structure/6rp8) (Crystal Structure of Ipilimumab Fab complexed with CTLA-4) as an example to demonstrate the process of MD simulations and free energy calculations. We proceeded with the 6RP8 in following steps

1. Protein modelling/ modeling protein missing residues
2. Molecular dynamics simulation with GROMACS
3. Free energy calculation with gmx_MMPBSA

**1. Protein modelling or modeling protein missing residues**

1.1 PDB 6RP8 was download from the <https://www.rcsb.org/structure/6rp8>.

1.2 The missing residues were manually analyzed in chimera by examining the sequence of 6RP8.

1.3 The missing residues were incorporated into the 6RP8 with the help of Modeler10.2 (<https://salilab.org/modeller/> ).

1.4 Two scripts were used: script-1.py and script-2.py.

**Script-1.py**

| from modeller import *  # Get the sequence of the 6rp8 PDB file, and write to an alignment file  code = '1i9r'  e = Environ()  m = Model(e, file=code)  aln = Alignment(e)  aln.append_model(m, align_codes=code)  aln.write(file=code+'.seq') |
| --- |

**Script-2.py**

| from modeller import *  from modeller.automodel import * # Load the AutoModel class  log.verbose()  env = Environ()  # directories for input atom files  env.io.atom_files_directory = ['.', '../atom_files']  a = LoopModel(env, alnfile = 'alignment.ali',  knowns = '6rp8', sequence = '6rp8_fill')  a.starting_model= 1  a.ending_model = 1  a.loop.starting_model = 1  a.loop.ending_model = 2  a.loop.md_level = refine.fast  a.make() |
| --- |

Notes:

1. To run the Modeller10.2 prior installation of Python is required.
2. The Modeller10.2 generated five best models on the basis of DOPE (Discrete Optimized Protein Energy). We chose the model with best DOPE score and proceed to MD Simulations.

**2. Molecular dynamics simulation with GROMACS**

2.1 The 6RP8 PDB we obtained from the Modeller does not have any protein molecules. If we were to use PDB directly from RCSB, then we need to strip out the crystal waters and other molecules. Such a procedure is not universally appropriate (i.e., the case of a water molecule bound to the active site). Here, we do not need crystal water or other molecular species, which are just crystallization co-solvents.

2.2 We prepare our system topology in two steps for the MD Simulation:

1. The protein topology is prepared with pdb2gmx.
2. Prepare the ligand topology using external tools (in the case of a ligand). Since we do not have a non-protein ligand in 6RP8, we do not run this step.

The command used is:

1. gmx pdb2gmx -f protein.pdb -o complex.gro

We were prompted to make 2 selections.

1. Force field
2. Water model


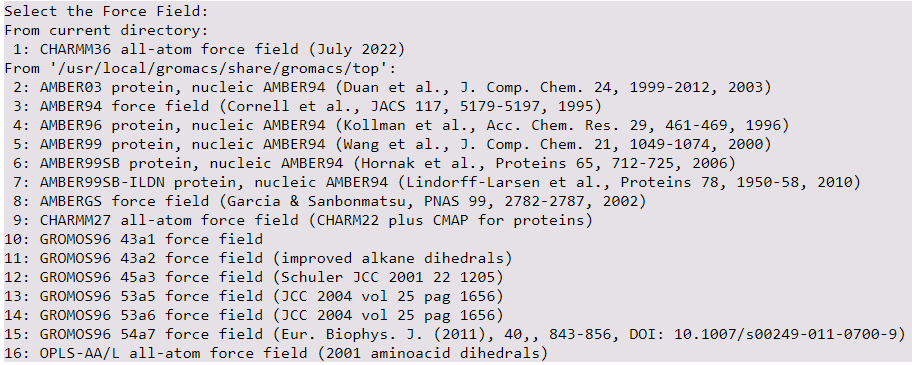


We have used no.9 force field CHARMM27 all-atom force field (CHARM22 plus CMAP for proteins) and SPC water model.

The files obtained after this are Topol.top, Complex.gro and Itp files for the 6RP8 complex.

1. In this step we defined the unit cell and filled it with water. For this step we run two commands.
2. gmx editconf -f complex.gro -o newbox.gro -bt dodecahedron -d 1.0
3. gmx solvate -cp newbox.gro -cs spc216.gro -p topol.top -o solv.gro

We obtaine two output files Newbox.gro and solv.gro, these files will be used for the next step of the simulation:

1. We now have a solvated system that contains a charged protein. The output of pdb2gmx indicates that the protein has a net charge (based on its amino acid composition). We can find this information at the last line of our [atoms] directive in *topol.top*. We add ions at this step to neutralize the system. The next step comprises the use of *grompp* to assemble a *.tpr* file, using *mdp* file. We use an *ions.mdp* file for running energy minimization, since they require the fewest parameters and are thus the easiest to maintain. The ions.mdp file is available in the folder of 6RP8 on Harvard Dataverse. We use the following commands:
2. gmx grompp -f ions.mdp -c solv.gro -p topol.top -o ions.tpr
3. gmx genion -s ions.tpr -o solv_ions.gro -p topol.top -pname NA -nname CL -neutral

We obtaine two output files ions.tpr and solv_ions.gro, these files will be used for the next step of the simulation:

1. Now that the system is assembled, binary input is create using grompp with em.mdp (file is available in the folder of 6RP8 simulation of dataverse) input parameter file:
2. gmx grompp -f em.mdp -c solv_ions.gro -p topol.top -o em.tpr

We obtaine two output files *em.tpr*, this file will be used for the next step of the simulation.

1. We then invoked the *mdrun* to carry out the EM
2. gmx mdrun -v -deffnm em
3. After the previous run the system reached at an energy minimum, the dynamics was initiated thereafter.
4. We proceed with *NVT* equilibration using nvt.mdp file (file is available in the folder of 6RP8 simulation of Harvard Dataverse). As we run the MD simulation on different temperatures *i.e.* 310K, 311K, 312K and 313K the changes are incorporated into this file.

a) gmx grompp -f nvt.mdp -c em.gro -r em.gro -p topol.top -n index.ndx -o nvt.tpr

b) gmx mdrun -deffnm nvt

We obtain the following files: nvt.tpr, nvt.trr, nvt.cpt and nvt.gro; these files will be used for the next step of the simulation.

1. Once the *NVT* simulation is complete, we proceed to *NPT* with npt.mdp file (file is available in the folder of 6RP8 simulation of Harvard Dataverse). Temperatures were changed in this file i.e 310K, 311K, 312K and 313K.
2. gmx grompp -f npt.mdp -c nvt.gro -t nvt.cpt -r nvt.gro -p topol.top -n index.ndx -o npt.tpr
3. gmx mdrun -deffnm npt

We obtain npt.tpr, npt.trr, npt.cpt and npt.gro, these files will be used for the next step of the simulation.

1. Upon completion of the two equilibration phases, the system is now well-equilibrated at the desired temperature and pressure. We now released the position restraints and run production MD for data collection. The process is just like we have seen before, and we make use of the checkpoint file (which in this case now contains preserve pressure coupling information) to grompp.
2. gmx grompp -f md.mdp -c npt.gro -t npt.cpt -p topol.top -n index.ndx -o md_0_10.tpr
3. gmx mdrun -deffnm md_0_10

We obtained Md_0_10.xtc, Md_0_10.gro, Md_0_10.cpt and Md_0_10.tpr. These files are the production files of MD simulation and used for the analysis part.

Recentering and Rewrapping Coordinates: As in any simulation conducted with periodic boundary conditions, molecules may appear "broken" or may "jump" back and forth across the box. To recenter the protein and rewrap the molecules within the unit cell to recover the desired rhombic dodecahedral shape, we invoked *trjconv*:

1. gmx trjconv -s md_0_10.tpr -f md_0_10.xtc -o dynamic-nopbc.xtc -pbc cluster

The output we obtained is dynamic-nopbc.xtc. This *xtc* file is the smaller file of the *md_0_10.xtc* files as it does not contain water molecules. We deliberately removed the water molecules and only selected protein to recenter and for the output.

We made an index file for 6RP8, the index file is necessary for a few analyses such as *HBonds* and also for the free energy calculations. In *HBonds* we need to specify the two groups between which we need the *HBonds*. For free energy calculations we need to specify the groups for the energy calculations.

1. gmx make_ndx -f em.gro -o index.ndx

We were provided with the option of making groups with this command. 6RP8 contains two parts: Ipilimumab Fab and CTLA-4. Ipilimumab Fab contains residues from 1-440, and CTLA-4 from 441-558. We separate both groups and make an index.ndx file.

1. Commands for the analysis of PDB 6RP8
2. RMSD: Root mean square deviation

gmx rms -s md_0_10.tpr -f dynamic-nopbc.xtc -o rmsd.xvg -tu ns

1. RMSF: Root mean square fluctuation

gmx rmsf -s md_0_10.tpr -f dynamic-nopbc.xtc -o rmsf.xvg -res

1. SASA: Solvent Accessible Surface Area

gmx sasa -s md_0_10.tpr -f dynamic-nopbc.xtc -o sasa.xvg

1. Radius of gyration

gmx gyrate -s md_0_10.tpr -f dynamic-nopbc.xtc -o gyrate.xvg

1. HBOND

gmx hbond -s md_0_10.tpr -n -index.ndx -f dynamic-nopbc.xtc -o hbond.xvg

**3. Free energy calculations with gmx_MMPBSA**

gmx_MMPBSA is written in Python 3.8 and combines functions from GROMACS and AmberTools to generate input files in a consistent/reproducible manner and perform end-state free energy calculations.

gmx_MMPBSA was installed and called along with GROMACS for the calculations of free energy.

**Workflow**

gmx_MMPBSA divides work into three major steps: (i) preparation, (ii) calculation, and (iii) visualization and analysis (Figure).

**Preparation:** The MMPSA.py calculation engine is used within gmx_MMPBSA to perform most of the calculations. The preparation process is focused on generating the topologies in Amber format in a consistent and reproducible way. In this step, the PDB structure files for the receptor and ligand are generated from the GROMACS structure + mass(db)files (i.e., *.tpr, *.pdb, or*.gro).

We used files from the MD simulation for the calculations of binding free energy.

Input files:

- Dynamics-nopbc.xtc (centered protein trajectory file)
- Topol.top
- Index.ndx
- Md_0_10.tpr

gmx_MMPBSA -O -i mmpbsa.in -cs md_0_10.tpr -ci index.ndx -cg 18 19 -ct dynamic-nopbc.xtc -cp topol.top -o FINAL_RESULTS_MMPBSA.dat -eo FINAL_RESULTS_MMPBSA.csv

**Free binding energy calculations:** In our studies we have calculated GB (Generalized Born) using *gmx_MMPBSA.*

**Results:** The results for 6RP8 are stored in the FINAL_RESULTS_MMPBSA.csv and can be accessed from Harvard Dataverse 6RP8 folder.

The results we obtained from the section Delta Energy Terms for 6RP8 at different temperatures are listed below. The free binding energy calculations were run for fever temperatures *i.e* 310K, 311K, 312K and 313K, with values obtained every 10 ns.

| 6RP8-RS1-200ns | | | |  |
| --- | --- | --- | --- | --- |
| 310K | 311K | 312K | 313K |  |
| -54.82 | -57.16 | -44.78 | -34.24 |  |
| -45.05 | -33.2 | -48.83 | -34.63 |  |
| -52.98 | -42.56 | -50.11 | -41.79 |  |
| -57.01 | -42.84 | -55.65 | -41.34 |  |
| -38.71 | -41.6 | -49.79 | -28.13 |  |
| -40.48 | -44.42 | -40.2 | -30.61 |  |
| -35.97 | -50.4 | -42.9 | -30.98 |  |
| -36.81 | -48.18 | -48.54 | -25.53 |  |
| -40.87 | -56.74 | -26.54 | -36.26 |  |
| -50.56 | -50.7 | -39.03 | -33.47 |  |
| -63.96 | -53.7 | -42.37 | -41.51 |  |
| -34.34 | -56.8 | -39.06 | -39.3 |  |
| -33.51 | -46.6 | -47.73 | -41.76 |  |
| -45.58 | -46.69 | -29.61 | -41.98 |  |
| -36.42 | -41.55 | -28.04 | -55.45 |  |
| -34.06 | -54.62 | -23.52 | -47.61 |  |
| -28.54 | -51.15 | -27.57 | -54.1 |  |
| -48.04 | -55.53 | -29.76 | -54.82 |  |
| -44.44 | -56.66 | -16.32 | -51.58 |  |
| -58.24 | -51 | -25.83 | -54.35 |  |
| -36.37 | -61.07 | -36.55 | -53.39 |  |
|  |  |  |  |  |
| -43.6552 | -49.6748 | -37.749 | -41.5633 | Averages |

An initial analysis indicates that, for this particular random seed, free binding energies increase at mild fever (311K), but then decrease with an increase in the temperature (by ~ 15%) from values obtained at 310K.
